# Immune landscape of distinct subtypes in urothelial carcinoma based on immune gene profile

**DOI:** 10.3389/fimmu.2022.970885

**Published:** 2022-08-08

**Authors:** Mou Peng

**Affiliations:** ^1^ Department of Urology, The Second Xiangya Hospital, Central South University, Changsha, China; ^2^ Key Laboratory of Diabetes Immunology (Central South University), Ministry of Education, National Clinical Research Center for Metabolic Disease, Changsha, China

**Keywords:** urothelial carcinoma, immune subtypes, tumor microenvironment, immunotherapy, biomarker

## Abstract

Immune checkpoint blockade (ICB) has become a promising therapy for multiple cancers. However, only a small proportion of patients display a limited antitumor response. The present study aimed to classify distinct immune subtypes and investigate the tumor microenvironment (TME) of urothelial carcinoma, which may help to understand treatment failure and improve the immunotherapy response. RNA-seq data and clinical parameters were obtained from TCGA-BLCA, E-MTAB-4321, and IMVigor210 datasets. A consensus cluster method was used to distinguish different immune subtypes of patients. Infiltrating immune cells, TME signatures, immune checkpoints, and immunogenic cell death modulators were evaluated in distinct immune subtypes. Dimension reduction analysis was performed to visualize the immune status of urothelial carcinoma based on graph learning. Weighted gene co-expression network analysis (WGCNA) was performed to obtain hub genes to predict responses after immunotherapy. Patients with urothelial carcinoma were classified into four distinct immune subtypes (C1, C2, C3 and C4) with various types of molecular expression, immune cell infiltration, and clinical characteristics. Patients with the C3 immune subtype displayed abundant immune cell infiltrations in the tumor microenvironment and were typically identified as “hot” tumor phenotypes, whereas those with the C4 immune subtype with few immune cell infiltrations were identified as “cold” tumor phenotypes. The immune-related and metastasis-related signaling pathways were enriched in the C3 subtype compared to the C4 subtype. In addition, tumor mutation burden, inhibitory immune checkpoints, and immunogenic cell death modulators were highly expressed in the C3 subtype. Furthermore, patients with the C4 subtype had a better probability of overall survival than patients with the C3 subtype in TCGA-BLCA and E-MTAB-4321 cohorts. Patients with the C1 subtype had the best prognosis when undergoing anti-PD-L1 antibody treatment. Finally, the immune landscape of urothelial carcinoma showed the immune status in each patient, and TGFB3 was identified as a potential biomarker for the prediction of immunotherapy resistance after anti-PD-L1 monoclonal antibody treatment. The present study provided a bioinformatics basis for understanding the immune landscape of the tumor microenvironment of urothelial carcinoma.

## Introduction

Urothelial carcinoma (UC) is the main pathological lesion of bladder cancer, which is the second most common urological malignancy in the United States, contributing to 83,730 estimated new cases and 17,200 estimated deaths in 2021 (1). Nonmuscle-invasive bladder cancer (NMIBC) is the predominant UC, and transurethral resection of the bladder tumor with intravesical bacillus calmette–guérin (BCG) vaccine treatment is the standard therapy for patients with NMIBC. For muscle-invasive bladder cancer (MIBC), the combination of radical cystectomy and neoadjuvant chemotherapy has become the standard therapy for MIBC (2). While metastasis and relapse occur, cisplatin-based chemotherapy is the first-line treatment for UC, and immune checkpoint inhibitors (ICIs) are recommended when chemotherapy or BCG fails or is intolerant. Nevertheless, the less than 30% objective response rate (ORR) of immune checkpoint inhibitors leads to limited clinical benefit (3, 4). Therefore, a thorough understanding of the tumor microenvironment (TME) of urothelial carcinoma is required to promote the antitumor response of urothelial carcinoma.

The response to ICI is thought to be based on a preexisting antitumor response, which is limited by adaptive immune resistance involving CD8^+^ T cells, CD4^+^, and B cells. The immune landscape of the tumor microenvironment of urothelial carcinoma, including the infiltration level of immune cells, cytokine secretion, and PD-L1 expression, is associated with the ICI-treated response of patients with UC and other cancers (5). Although the tumor microenvironment is classified into three phenotypes (immune-excluded, immune-desert, and immune-inflamed phenotypes) (6), understanding the sensitivity and resistance to ICI as well as elucidating the detailed landscape of the tumor microenvironment are required for comprehending the distinct clinical outcomes of immunotherapy. The immune-inflamed phenotype, also known as a “hot” tumor, is characterized by immune cell infiltration and potential response to immunotherapy (7). Nevertheless, not all patients with a “hot” tumor phenotype based on this classification have a complete response to ICI. Therefore, exploring novel classifications and the mechanisms of cell populations or signaling interactions may provide preferential targets to overcome this resistance.

The purpose of the present study was to characterize the immune landscape of urothelial carcinoma. Regarding the expression profile of immune-related genes, we distinguished four immune subtypes and seven coexpression modules of urothelial carcinoma. Each immune subtype corresponded to different molecular expression levels, immune cell infiltration, and clinical characteristics. Ultimately, the immune landscape of urothelial carcinoma was characterized by the immune status of each patient. Our findings provide an early exploration and bioinformatics basis for understanding the immune landscape of the tumor microenvironment of urothelial carcinoma, providing new knowledge of the ICI-related antitumor response to immunotherapy.

## Methods

### Expression profiling and data preprocessing

TCGA-BLCA FPKM gene expression data and corresponding clinical information of 408 patients with bladder urothelial carcinoma from UCSC Xena (training cohort, https://xenabrowser.net/datapages), the FPKM gene expression data of 476 patients with nonmuscular invasive bladder urothelial carcinoma from E-MTAB-4321 (8) (validation cohort 1, https://www.ebi.ac.uk/arrayexpress/experiments/E-MTAB-4321), and the gene expression data of 348 patients with metastatic urothelial carcinoma (mUC) from IMVigor210 (validation cohort 2, http://research-pub.gene.com/IMvigor210CoreBiologies) were collected (9). In total, 1793 immune-related genes from the ImmPort database (https://www.immport.org/shared/genelists), including cytokines, cytokine receptors, chemokines, chemokine receptors, TCR signaling pathway, BCR signaling pathway, antigen presentation, natural killer cell cytotoxicity, TGFβ family members, TGFβ receptors, TNF family members, TNF receptors, interferons, interferon receptors, interleukins and interleukin receptors, were obtained.

The transcriptional sequence gene expression data from TCGA-BLCA, E-MTAB-4321, and IMVigor210 were transformed into transcripts per million (TPM) for subsequent analysis. The data of nontransitional cell carcinoma samples from TCGA-BLCA were filtered out. The org.Hs.eg.db package in R was used to map gene names and annotate them into gene symbols. Duplicated tumor samples and patients with missing clinical information were eliminated. Finally, 405 patients with 1725 immune-related genes from TCGA-BLCA, 476 samples with 1320 immune-related genes from E-MTAB-4321, and 348 samples with 1350 immune-related genes from IMVigor210 were obtained. The gene expression data were transformed using log2(TPM+1).

### Classification and validation of immune subtypes

After preprocessing the data of immune-related genes, the partitioning around medoids (PAM) clustering algorithm with 1000 bootstraps and the 1-Pearson correlation as a distance metric for clustering were performed using “ConsensusClusterPlus” R package, each containing 80% of patients in the training cohort. Patients were clustered from rank 2 to 9, and the optimal k value was selected by consensus matrix, consensus CDF and delta area. The classification of immune subtypes was subsequently validated in two independent cohorts (E-MTAB-4321 and IMVigor210) with the same parameters. According to sex, race, and tumor stage, the frequency of immune subtypes was calculated. The overlap between the immune subtypes we generated and immune subtypes from the previous report by Thorsson was obtained (10).

### Status of tumor mutation burden in each immune subtype

The somatic mutation “maf” file of TCGA-BLCA was downloaded from UCSC Xena (https://xenabrowser.net/) (11). The tumor mutation burden (TMB) in bladder urothelial carcinoma was analyzed by the Maftools package in R. The landscape of TMB was obtained, and the total TMB per megabyte was compared according to distinct immune subtypes.

### Distributions of tumor-infiltrating immune cells and tumor microenvironment signatures of immune subtypes

Regarding the four immune subtypes, the relationships of tumor-infiltrating immune cells, molecular signatures, immune checkpoints (ICPs), and immunogenic cell death (ICD) modulators were assessed. The scores of 29 types of tumor-infiltrating immune cells and 29 tumor microenvironment signatures in bladder urothelial carcinoma were calculated by the ssGSEA method (12). Consistent with a previous publication (13), activated CD4**^+^** T cells, activated CD8**^+^** T cells, central memory CD4**^+^** T cells, central memory CD8**^+^** T cells, effector memory CD4**^+^** T cells, effector memory CD8**^+^** T cells, type 1 T helper cells, type 17 T helper cells, activated dendritic cells, CD56^bright^ natural killer cells, natural killer cells, and natural killer T cells were defined as “antitumor immunity”. Regulatory T cells, type 2 T helper cells, CD56^dim^ natural killer cells, immature dendritic cells, macrophages, MDSCs, neutrophils, and plasmacytoid dendritic cells were considered as “protumor suppression”. The correlation between antitumor immunity and protumor suppression was analyzed using the Pearson correlation method

To furtherly validate the enrichment pattern of immune cells in each immune subtype, we chose multiple algorithms to calculate the scores of immune cells. Data were downloaded from Timer2.0, including the estimation results from TIMER, CIBERSORT, quanTIseq, xCell, MCP-counter and EPIC methods (14).

### Gene Set Enrichment Analysis

In TCGA-BLCA database, GSEA was then performed to detect the gene sets that were enriched in the gene rank between the C3 and C4 immune subtypes to identify potential Kyoto Encyclopedia of Genes and Genomes (KEGG) signaling pathways of urothelial carcinoma. The c2.cp.kegg.v7.5.symbols.gmt annotation file in the Molecular Signatures Database (MSigDB) was selected in GSEA version 4.1.0. The following parameters were set: 1000 permutations; the collapse/remap to gene symbols was “No_collapse”; and the permutation type was “phenotype”. GSEA was run, and the cutoff criteria were as follows: |normalized enrichment scores (NES)| > 1.0 and nominal p < 0.05. Immunity-related gene sets with significant enrichment are displayed as enrichment plots.

### Weighted Correlation Network Analysis

The immune-related gene expression profile of TCGA-BLCA was performed by the WGCNA package in R to identify the gene coexpression modules (15). The relationship between gene coexpression modules and clinical features was calculated by the eigengene module. The clinical features included the status and time of overall survival (OS). The OS-associated modules were functionally annotated, and Gene Ontology (GO) and KEGG pathway enrichment analyses were performed using the clusterProfiler (16) and DOSE packages (17) in R. The hub genes in modules of interest were identified by gene connectivity.

### Prediction of immunotherapy response according to hub genes

The hub genes obtained from modules of interest were furtherly evaluated in the IMVigor210 cohort, in which mUC patients have undergone anti-PD-L1 monoclonal antibody. Kaplan-Meier plot was performed to evaluate the prognostic value of hub genes. The expression levels of hub genes between the response group and the non-response group were analyzed. The “anova” method was used to calculate the p-value for statistical analysis.

### Characterization of the immune landscape

The Monocle package in R was used to perform dimensionality reduction analysis based on graph learning (18). Dimension reduction was initialized with a tobit distribution and the discriminative dimensionality reduction with trees (DDRTree) algorithm. The maximum number of principal components was selected as two, and the normalized method was set by log. Finally, the trajectory analysis of the Monocle package was utilized to display the distribution of patients with multiple colors corresponding to the classification and status of immune subtypes.

## Results

### Identification of immune subtypes in urothelial carcinoma

**Figure 1:**
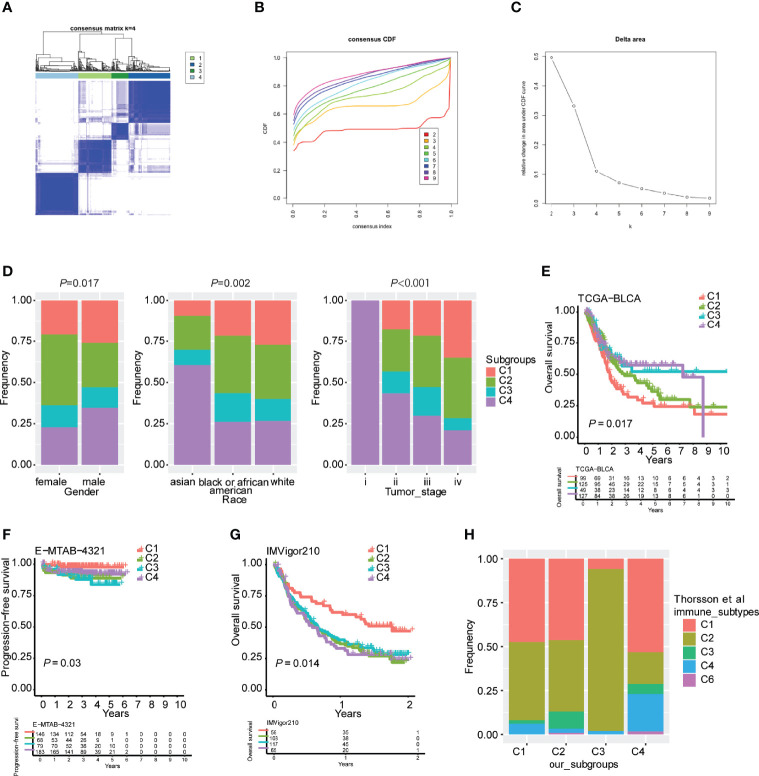
Robust classification of distinct immune subtypes of bladder urothelial carcinoma. **(A)** Consensus clustering analysis of bladder urothelial carcinoma patients. **(B)** Cumulative distribution function (CDF) curve of the training cohort. **(C)** CDF delta area curve of the training cohort. **(D)** Distribution of immune subtypes according to sex, race, and tumor stage. **(E)** Kaplan plot of overall survival in TCGA-BLCA cohort. **(F)** Kaplan plot of progression-free survival in the E-MTAB-4321 cohort. **(G)** Kaplan plot of overall survival in the IMVigor210 cohort. **(H)** The overlap between our four immune subtypes and Thorsson’s six subtypes.

The antitumor immune response is heterogeneous in distinct immune subtypes of bladder urothelial carcinoma responding to immune checkpoint inhibitors. In the present study, consensus clustering analysis was performed, and k=4 was selected to cluster patients with bladder urothelial carcinoma into four stable clusters according to consensus CDF and delta area (**Figure 1A-C**). We designated these four immune subtypes as C1, C2, C3, and C4. The immune subtype distribution was significantly correlated with sex, race, and tumor stage (**Figure 1D** and **Supplementary materials 1**). C3 and C4 subtypes were associated with better overall survival, whereas the C1 subtype was associated with the worst overall survival in TCGA-BLCA cohort (**Figure 1E**). Furthermore, the immune subtypes were also associated with overall survival in the E-MTAB-4321 and IMVigor210 cohorts, which was inconsistent with the results from the TCGA-BLCA cohort. In early-stage nonmuscular invasive bladder urothelial carcinoma, the C2 and C3 subtypes were associated with poorer progression-free survival, whereas the C1 and C4 subtypes were associated with better progression-free survival (**Figure 1F**). In the IMVigor210 cohort, patients with metastatic urothelial carcinoma received anti-PD-L1 immunotherapy, and the C1 subtype was associated with better overall survival of patients (**Figure 1G**).

To validate the reliability of these immune subtypes for bladder urothelial carcinoma, we investigated the overlap between the four immune subtypes we generated and six pancancer subtypes that have been previously reported (C1-C6), of which patients with bladder urothelial carcinoma were clustered into five pancancer subtypes (C1-4 and C6). We observed that the lymphocyte-depleted immune subtype (C4) in Thorsson’s publication mostly overlapped with the C4 subtype in the present study, whereas the IFN-γ immune subtype (C2) in Thorsson’s publication overlapped with the C3 subtype in the present study, which correlated with better overall survival (**Figure 1H**). The inflammatory immune subtype (C3) in Thorsson’s publication was more enriched in the C2 subtype of the present study (**Figure 1H**). These findings indicated that the robust classification of immune subtypes could be utilized for predicting survival possibilities in different urothelial carcinoma cohorts.

### Tumor mutational burden in different immune subtypes

**Figure 2:**
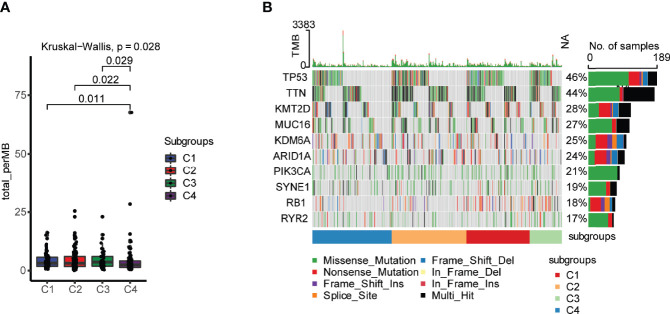
Landscape of tumor mutational burden (TMB) in four different immune subtypes. **(A)** TMB in four different immune subtypes of bladder urothelial carcinoma. **(B)** Mutation status of the top 10 immune genes with genomic alterations in the four different immune subtypes of bladder urothelial carcinoma.

Tumor mutational burden (TMB) is considered a predictive biomarker for immunotherapeutic efficacy (19, 20). We used the mutect2-processed mutation dataset of TGCA-BLCA cohort to calculate the TMB and compared it to each subtype. The C4 subtype had the lowest TMB in comparison with the other immune subtypes (**Figure 2A**). The mutation status of the top 10 immune genes with genomic alterations was identified in the four different immune subtypes of bladder urothelial carcinoma, indicating the distribution of the tumor neoantigens in four different immune subtypes of bladder urothelial carcinoma (**Figure 2B**). The **Supplementary Figure 1** also detailedly displayed the different proportions of top10 high mutated genes in each immune subtypes.

### TME expression pattern of immune molecules according to immune subtypes

**Figure 3:**
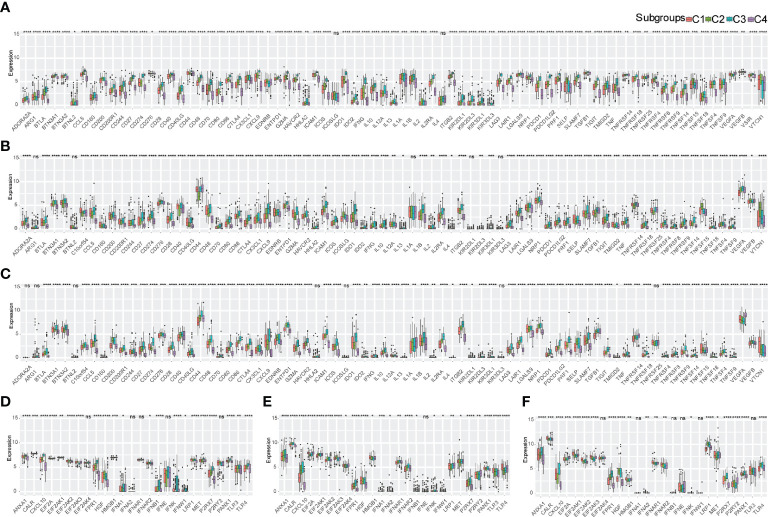
Expression pattern of immune molecules in the TME of bladder urothelial carcinoma according to immune subtypes. **(A-C)** Differential expression of immune checkpoints in the four immune subtypes in TCGA-BLCA cohort **(A)**, E-MTAB-4321 cohort **(B)**, and IMVigor210 cohort **(C)**. **(D-F)** ICD expression in the four immune subtypes in TCGA-BLCA cohort **(D)**, E-MTAB-4321 cohort **(E)**, and IMVigor210 cohort **(F)**. ns, p ≥ 0.05; *p < 0.05; **p < 0.01; ***p < 0.001; and ****p < 0.0001.

The expression of immune checkpoints (ICPs) and immunogenic cell death (ICD) modulators in the TME affects the activation of the antitumor immune response and may play critical roles in the antigen-presenting process and cytotoxicity (21). We analyzed the expression of ICPs and ICD modulators in four different immune subtypes of bladder urothelial carcinoma. In total, 78 immune checkpoints were detected in the three cohorts with 72 genes in TCGA-BLCA (**Figure 3A**), 72 genes in E-MTAB-4321 (**Figure 3B**), and 71 genes in the IMVigor210 cohort (**Figure 3C**) differentially expressed in the four immune subtypes. Costimulatory checkpoints (such as PD-1, CTLA4, and PD-L1) and coinhibitory checkpoints (such as ICOS, CD28, and 4-1BB) were highly expressed in the C3 subtype but expressed at low levels in the C4 subtype. Immunostimulatory danger-associated molecular patterns (DAMPs) (such as ANXA1, CALR, and HMGB1) and cytokines (such as IFN and CXCL10) were used to evaluate the immunogenicity of cancer cell death. In total, 26 immunogenic cell death modulators were evaluated in the three cohorts with 21 ICD modulators in TCGA-BLCA cohort, 24 ICD modulators in the E-MTAB-4321 cohort, and 20 ICD modulators in the IMVigor210 cohort differentially expressed in the four different immune subtypes. The C3 subtype had high immunogenicity, and the C4 subtype had low immunogenicity in the three cohorts (**Figure 3D–F**). Taken together, these findings indicated that there were different expression levels of immune checkpoints and ICD modulators in different immune subtypes. Thus, immune subtypes may be effective predictors of immunotherapy response.

### Infiltration of immune cells and tumor microenvironment signatures of immune subtypes

**Figure 4:**
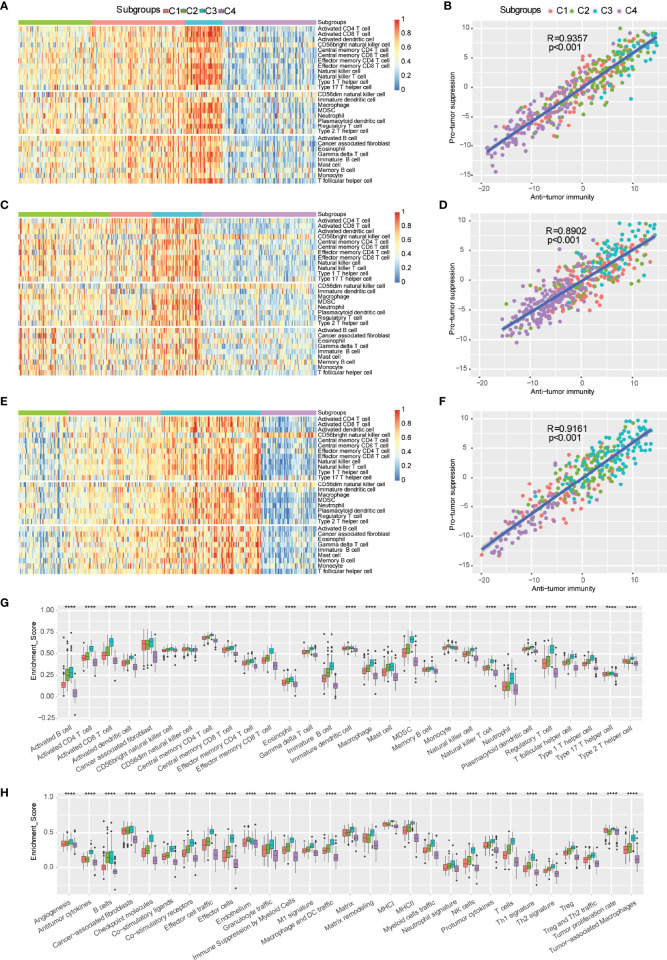
Distributions of tumor-infiltrating immune cells and molecular signatures of immune subtypes. **(A-F)** Heatmap and correlation analysis between immune subtypes and tumor-infiltrating cells in TCGA-BLCA **(A, B)**, E-MTAB-4321 **(C, D)**, and IMvigor210 **(E, F)**. **(G)** Distribution of 29 immune cells in distinct immune subtypes. **(H)** Scores of 29 molecular signatures in distinct immune subtypes. ns, **p < 0.01; ***p < 0.001; and ****p < 0.0001.

The infiltration of immune cells and tumor microenvironment signatures reflect the status of the immune response in the TME and influence the treatment effect of immunotherapy. We further used the ssGSEA method to score 29 tumor-infiltrating immune cells and characterize the immune cell components in the four distinct immune subtypes. Antitumor immunity was involved in 17 types of immune cells, such as activated CD4^+^ T cells and activated CD8^+^ T cells, whereas protumor suppression was involved in 8 types of immune cells, such as MDSCs and regulatory T cells. The highest percentage of immune cell infiltration occurred in the C3 subtype, but the lowest percentage of immune cell infiltration occurred in the C4 subtype according to the three independent cohorts. Moderate infiltration of immune cells occurred in C1 and C2 subtypes (**Figures 4A, C, E**). The C4 immune subtype had a larger proportion of patients with NMIBC (E-MTAB-4321) than MIBC (TCGA-BLCA) and mUC (IMVigor210). Furthermore, multiple algorithms revealed the same trend of immune cell infiltration in each immune subtype (**Supplementary Figure 2**).

The correlation analysis indicated that antitumor immunity was positively correlated with protumor suppression (**Figures 4B, D, F**). Furthermore, the scores of antitumor immunity-related cells (such as activated B cells, activated CD4^+^ T cells, activated CD8^+^ T cells, activated dendritic cells, and CD56^bright^ NK cells) and protumor suppression-related cells (such as MDSCs, regulatory T cells, type 2 T helper cells, and neutrophils) were more enriched in the C3 subtype compared to the other immune subtypes, and they were less enriched in the C4 subtype (**Figures 4G, H**). Therefore, the C3 subtype was considered an immunological “hot” tumor, whereas the C4 subtype was considered an immunological “cold” tumor in TCGA-BLCA cohort, which was consistent with the trends of the E-MTAB-4321 cohort and IMVigor210 cohort (**Supplementary Figure 3**). In addition, to further distinguish “hot” tumors, we proposed a new concept of “warm” tumors, which ranged between “hot” tumors and “cold” tumors. The C1 and C2 subtypes were identified as “warm” tumors. The robust classification of immune subtypes of bladder urothelial carcinoma will help guide immunotherapy to suitable patients. mUC patients with the C1 immune subtype, belonging to “warm” tumors, showed a significant improvement after anti-PD-L1 immunotherapy. We subsequently observed that the differences in 29 tumor microenvironment signatures were statistically significant in the four ISs. The C3 subtype had the highest scores of antitumor immune infiltration (MHCI, MHCII, costimulatory ligands, costimulatory receptors, T cells, effector cells, effector cell trafficking, NK cells, B cells, M1 signature, Th1 signature, and antitumor cytokines), protumor immune infiltration (Treg, Treg and Th2 traffic, neutrophil signature, granulocyte traffic, immune suppression by myeloid cells, macrophages, DC traffic, Th2 signature, and protumor cytokines), angiogenesis, and fibroblasts (CAFs, matrix, and matrix remodeling), thereby presenting the “hot” immune phenomenon, whereas the C4 subtype had the lowest scores of these signatures, thereby displaying the “cold” immune phenomenon. Furthermore, there was also heterogeneity between the C1 and C2 subtypes. Compared to the C2 subtype, the C1 subtype had more trends of antitumor cytokines, checkpoint molecules, effector cell traffic, effector cells, M1 signature, MHCI, NK cells, Th1 signature, Treg/Th2 traffic, and tumor proliferation rate. Thus, these findings suggested that the C1 subtype indicates patients suitable for anti-PD-L1 monoclonal antibody (mAb) immunotherapy.

### Signaling pathway enrichment using GSEA

**Figure 5:**
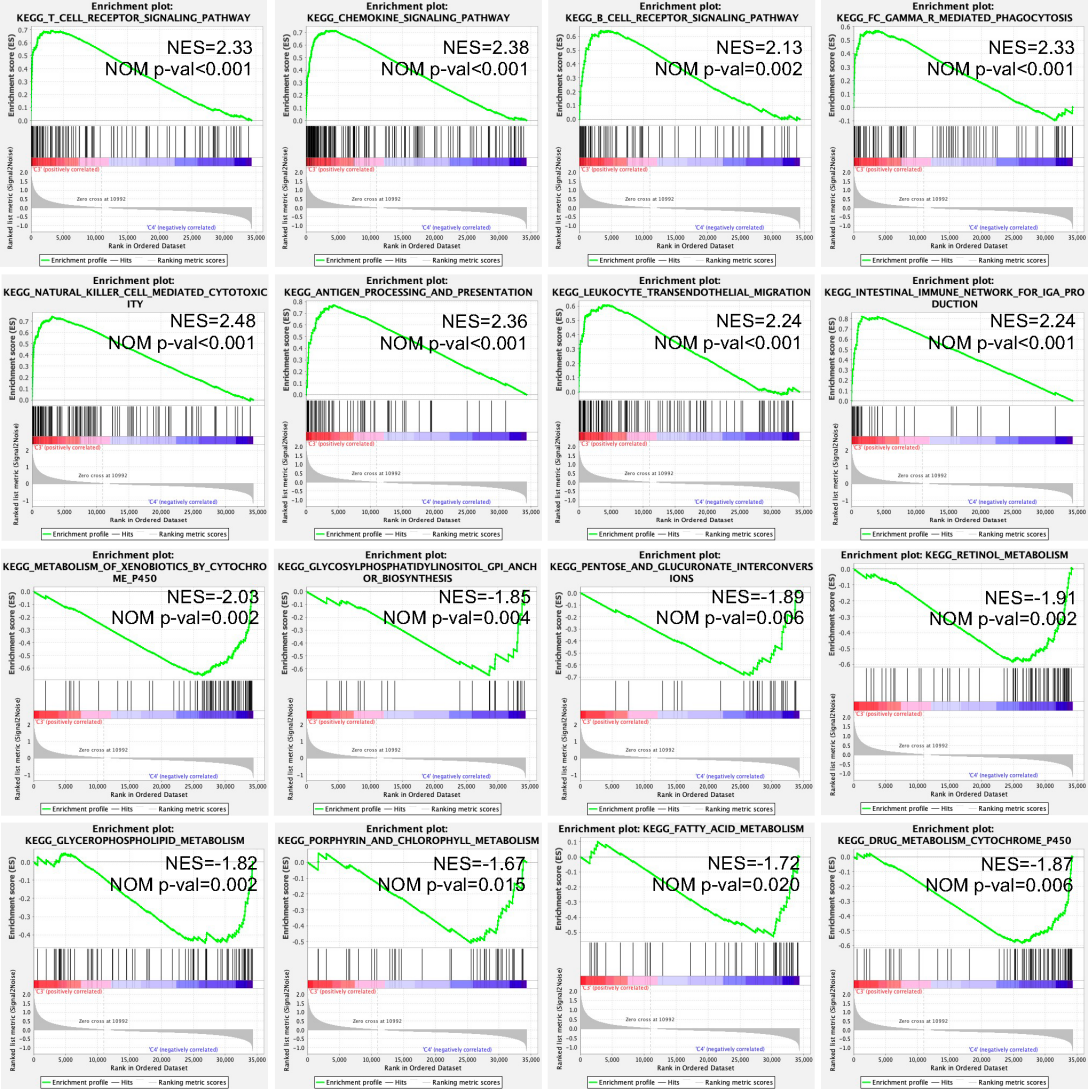
Enriched KEGG signaling pathways in the C3 and C4 immune subtypes using the GSEA method. NES, normalized enrichment score; NOM P-val, nominal p value; KEGG, Kyoto Encyclopedia of Genes and Genomes; and GSEA, Gene Set Enrichment Analysis.

The expression matrix of the C3 and C4 immune subtypes was analyzed by the GSEA method. In total, 60 signaling pathways were enriched in the C3 immune subtype, including metastasis-related signaling pathways and immunity-related signaling pathways, such as “T cell receptor signaling pathway”, “chemokine signaling pathway”, “B cell receptor signaling pathway”, “FC gamma R mediated phagocytosis”, “natural killer cell-mediated cytotoxicity”, “antigen processing and presentation”, “leukocyte transendothelial migration”, and “intestinal immune network for IgA production”, whereas 15 metabolism-related signaling pathways were enriched in the C4 immune subtype, including “metabolism of xenobiotics by cytochrome P450”, “glycosylphosphatidylinositol GPI anchor biosynthesis”, “pentose and glucuronate interconversions”, “retinol metabolism”, “glycerophospholipid metabolism”, “porphyrin and chlorophyll metabolism”, “fatty acid metabolism”, and “drug metabolism cytochrome P450” (**Figure 5**).

### Further screening of suitable patients for immunotherapy

**Figure 6:**
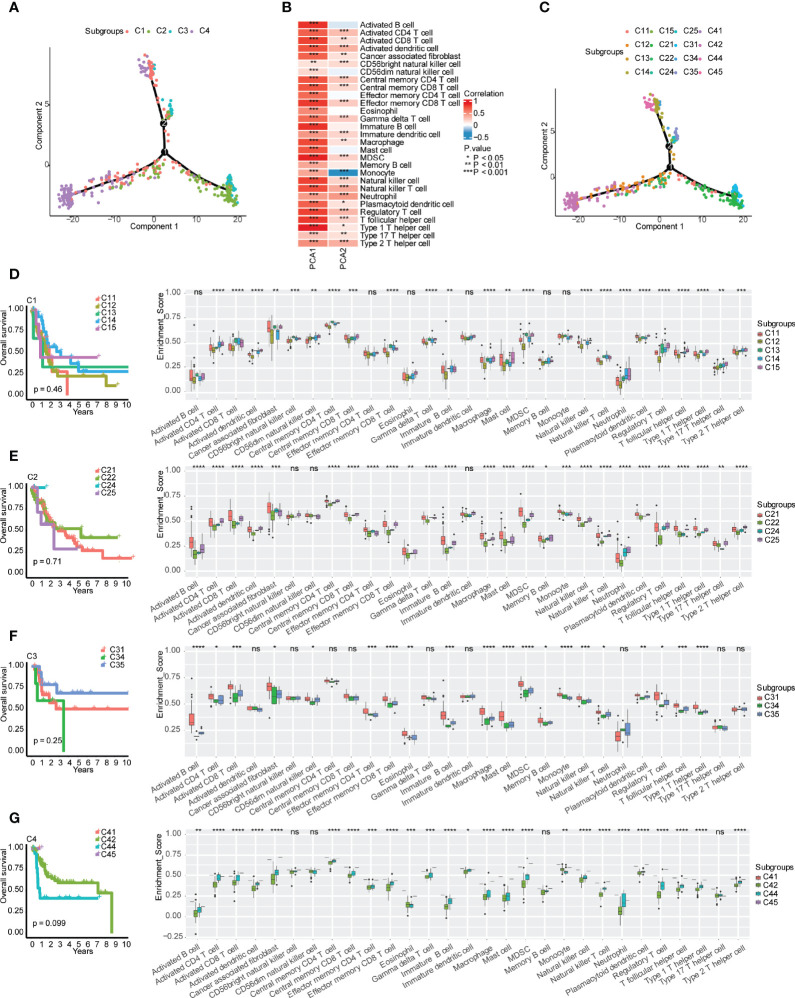
Immune gene-related landscape of bladder urothelial carcinoma. **(A)** Distribution of patients after reduced dimension. **(B)** Correlations between principal component analysis and immune cells. **(C)** Distribution of patients representing the status of each immune subtype. **(D–G)** Stratification analysis of immune subtypes. **(D)** Prognostic value and immune cell components in different subgroups of the C1 subtype. **(E)** Prognostic value and immune cell components in different subgroups of the C2 subtype. **(F)** Prognostic value and immune cell components in different subgroups of the C3 subtype. **(G)** Prognostic value and immune cell components in different subgroups of the C4 subtype. , ns, p ≥ 0.05; *p < 0.05; **p < 0.01; ***p < 0.001; and ****p < 0.0001.

According to the expression of immune-related genes, the immune landscape of bladder urothelial carcinoma was further characterized and visualized to expand the screen of suitable patients for immunotherapy (**Figure 6A**). Principal component 1 (PCA1) was positively correlated with all 29 types of immune cells, while the correlation between principal component 2 (PCA2) and immune cells had more diversity (**Figure 6B**). PCA2 was positively correlated with activated CD4^+^ T cells, activated CD8^+^ T cells, activated dendritic cells, cancer-associated fibroblasts, CD56^bright^ natural killer cells, central memory CD4^+^ T cells, central memory CD8^+^ T cells, effector memory CD56^bright^ cells, gamma delta T cells, immature dendritic cells, macrophages, MDSCs, natural killer cells, natural killer T cells, neutrophils, plasmacytoid dendritic cells, regulatory T cells, T follicular helper cells, type 1 T helper cells, type 2 T helper cells, and type 17 T helper cells, but PCA2 was negatively correlated with monocytes (**Figure 6B**). Further clustering analysis indicated that there were 5 subgroups in the C1 subtype, 4 subgroups in the C2 subtype, 3 subgroups in the C3 subtype, and 4 subgroups in the C4 subtype (**Figure 6C**). However, survival analysis of these subgroups in each immune subtype indicated no significant difference in prognosis in each immune subtype (**Figures 6D–G**). The immune cell infiltration and tumor microenvironment signature analysis indicated the heterogeneity in each immune subtype, of which C14 and C21 in the “warm” tumor immune subtype and C31 in the “hot” tumor immune subtype may be the second priority of potential patients for immunotherapy (**Supplementary Figure 4**).

### Identification of biomarkers for evaluating the effectiveness of immunotherapy

**Figure 7:**
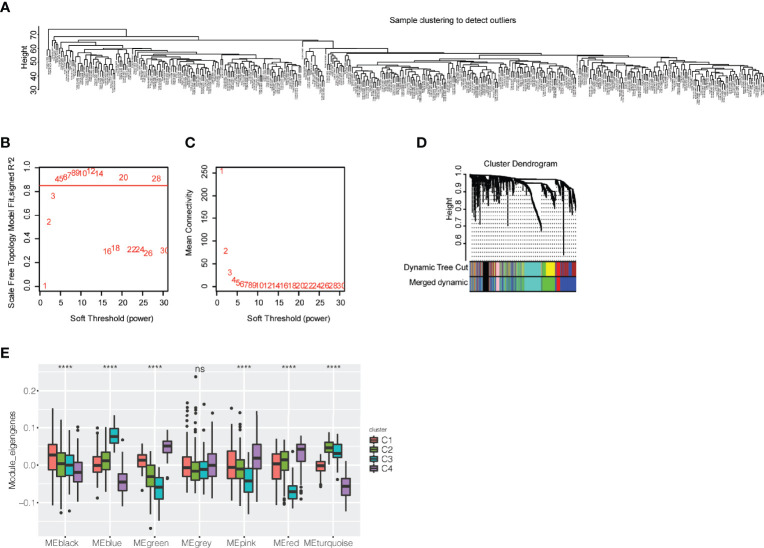
Exploration of coexpression modules of immune-related genes. **(A)** Clustering of patients with bladder urothelial carcinoma based on the expression profile of immune-related genes. **(B)** Scale-free topology model for the identification of multiple soft thresholds. **(C)** Mean connectivity for multiple soft thresholds. **(D)** Cluster dendrogram and module colors. **(E)** Module eigengenes of seven modules in the immune subtypes of bladder urothelial carcinoma ns, p ≥ 0.05; and ****p < 0.0001.

WGCNA was utilized to identify immune-related gene coexpression modules based on immune-related gene expression profiles, and the sample clustering is displayed in **Figure 7A**. We found that a soft threshold of 4 was good for the scale-free topology model, and the expression matrix was transformed into an adjacency matrix (**Figures 7B, C**). The cutreeDynamic function was then used to identify modules with the setting of at least 30 genes. After calculation of module eigengenes, we set the threshold of module eigengene dissection as 0.30, and 7 coexpression modules were finally obtained for further analysis (**Figure 7D**). We compared the module eigengenes of the four immune subtypes, and there were significant differences in the blue, black, green, pink, red, and turquoise modules (**Figure 7E**). The C3 subtype had the highest module eigengenes in the blue module, whereas the C4 subtype had the highest module eigengenes in the green, pink, and red modules. The C1 subtype, which may benefit from immunotherapy, showed the highest module eigengenes in the black module.

**Figure 8:**
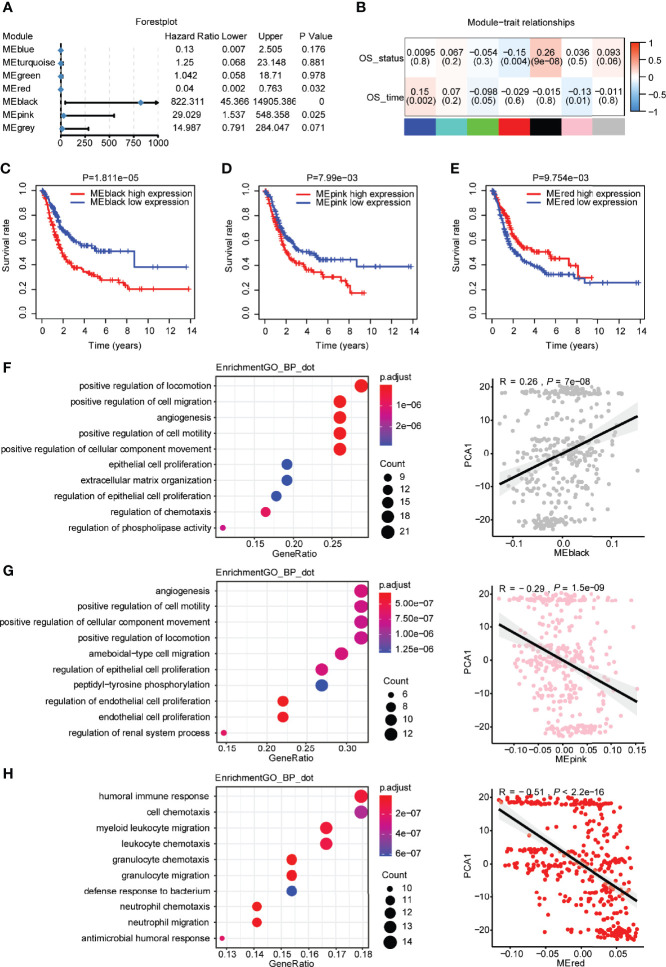
Identification of hub genes of bladder urothelial carcinoma based on immune genes. **(A)** Forest plot of univariate Cox analysis of seven modules of bladder urothelial carcinoma. **(B)** The degree of correlation between different modules and survival information is shown. **(C)** Prognostic value of the black modules with the median as a cutoff. **(D)** Prognostic value of the pink modules with the median as a cutoff. **(E)** Prognostic value of the red modules with the median as a cutoff. **(F)** Dot plot of the top 10 biological processes in terms of the black module. Correlation between the black module and principal component 1. **(G)** Dot plot of the top 10 biological processes in terms of the pink module. Correlation between the pink module and principal component 1. **(H)** Dot plot of the top 10 biological processes in terms of the red module. Correlation between the red module and principal component 1.

Regarding the module eigengenes, survival analysis indicated that the black module was significantly associated with the status of overall survival, while the red and pink modules were associated with the time of overall survival (**Figures 8A, B**). High scores of black and pink module eigengenes were correlated with a poor prognosis, whereas a low score of red module eigengenes was correlated with a poor prognosis (**Figures 8C–E**). Furthermore, GO analysis revealed that the black module with genes enriched in the following biological processes was positively correlated with PCA1 (**Figure 8F**): positive regulation of locomotion, positive regulation of cell migration, angiogenesis, epithelial cell proliferation, and extracellular matrix organization. Similarly, the pink module with genes enriched in the following biological processes was negatively correlated with PCA1 (**Figure 8G**): angiogenesis, positive regulation of cell motility, regulation of epithelial cell proliferation, regulation of endothelial cell proliferation, and endothelial cell proliferation. The red module with genes enriched in myeloid leukocyte migration, granulocyte migration, and neutrophil migration was negatively correlated with PCA1 (**Figure 8H**). The cellular components and molecular functions of the GO and KEGG signaling pathways are displayed in **Supplementary Figure 5**. The C1 immune subtype had the best improvement in overall survival after immunotherapy, while the black and pink modules had high module eigengene scores in the C1 subtype. Therefore, hub genes in the black and pink modules may be a potential feature to consider patients with bladder urothelial carcinoma for immunotherapy. Subsequently, we obtained three hub genes with more than 20% gene significance and 80% intramodular connectivity in the black module: fibroblast growth factor receptor 1 (FGFR1), annexin A6 (ANXA6), and transforming growth factor beta-3 (TGFB3). These three genes were furtherly identified as potential biomarkers for the prediction of antitumor immune response. According to response evaluation criteria in solid tumors (RECIST) (22), patients were divided into two groups: stable disease (SD)/progressive disease (PD), and complete response (CR)/partial response (PR). We found that high expression of TGFB3, which displayed in the SD/PD group, was associated with a poor prognosis of metastatic UC undergoing anti-PD-L1 monoclonal antibody treatment (**Supplementary Figure 6**).

## Discussion

Previous studies have classified patients with bladder cancer into different subtypes using different methods. Tang et al. revealed that the highest immune cell infiltration was positively associated with good overall survival (23). Wang et al. also indicated that the immune-hot phenotype may benefit from immunotherapy (24). However, only some patients with an immune-hot phenotype respond to immunotherapy. Elucidation of the heterogeneity of the tumor microenvironment in the immune-hot phenotype is required for more understanding.

In the present study, four distinct immune subtypes were identified based on the expression profile of immune-related genes. Based on TCGA-BLCA cohort, patients with C4 and C3 subtypes had better overall survival than those with C1 and C2 subtypes, suggesting that this robust classification is a good biomarker for bladder urothelial carcinoma. The frequency reduction of the C3 and C4 subtypes with a good prognosis was followed by improved tumor staging. Thorsson et al. classified 6 immune clusters of 33 pancancers and suggested that they are relevant to prognosis (10). The distribution of 5 immune clusters, except for C5, was observed in bladder urothelial carcinoma. The C1 (wound healing) and C2 (IFN-γ) subtypes with a good prognosis were overlapped in the C3 subtype. Additionally, our classification was also robust and prognostically relevant in patients with nonmuscular invasive bladder urothelial carcinoma and patients with metastatic urothelial carcinoma undergoing anti-PD-L1 mAb immunotherapy. Our results revealed that bladder urothelial carcinoma is associated with this classification, which was different from previous immune subtypes (25). This classification provided more information for selecting suitable patients for immunotherapy.

Regarding the high tumor mutation burden of bladder urothelial carcinoma, bladder cancer is a type of malignant tumor suitable for immunity-based therapy, such as intravesical BCG and ICI. Thus, patients with bladder urothelial carcinoma have different immune responses to ICIs due to tumor heterogeneity and dynamic alterations in the immune signature in the tumor microenvironment. Our classification of immune subtypes revealed that the C4 subtype was a “cold” phenotype, which was infiltrated with CD56^bright^ natural killer cells and CD56^dim^ natural killer cells in urothelial carcinoma, representing a noninflammatory tumor microenvironment. The C3 subtype was an inflammatory phenotype with the highest infiltration level of antitumor immune cells (e.g., activated CD4^+^ T cells and activated CD8^+^ T cells) and suppressive immune cells (e.g., Tregs, MDSCs, and neutrophils). Interestingly, the C4 immune subtype had better overall survival than the C3 immune subtype in TCGA-BLCA cohort. Previous studies have found that the cluster with the lowest immune and stromal scores has a good prognosis, which may be explained by the high immune infiltration of naive CD4^+^ T cells (25). Another study has reported a cluster with the lowest immune cell infiltration, and the best prognosis of this cluster tends to be younger with low TNM Classification of Malignant Tumors (TNM) and clinicopathological stage (26). GSEA found that there were immunity-related signaling pathways, cancer-related pathways, and metastatic pathways in the C3 immune subtype that may be associated with a poor prognosis, whereas metabolism-related pathways were enriched in the C4 subtype (**Supplementary materials 2**). Furthermore, metabolism-related pathways and DNA repair-related pathways were enriched in the C1 subtype, and immunity-related signaling pathways and metastatic pathways were enriched in the C2 subtype (**Supplementary Materials 3**). Moreover, we defined the C1 and C2 subtypes as “warm” phenotypes, displaying limited immune cell infiltration between the “cold” phenotype and the “hot” phenotype, of which the C1 subtype has a more effective immune response for anti-PD-L1 mAb than the C2 subtype.

Furthermore, the differential expression of ICPs and ICD modulators affects the immune status of the tumor microenvironment. There were different potential mechanisms of immune evasion in the four distinct immune subtypes in the present study, which may require different therapeutic treatments. A cancer vaccine may be suitable for the recruitment of more lymphocytes to the tumor microenvironment in the C4 immune subtype. Patients with C2 and C3 immune subtypes may benefit from combination immunotherapy therapy due to the high expression levels of coinhibitory checkpoints (such as PD-L1, PD1, and CTLA4) and immunosuppressive cytokines (such as IL6, IL10, and TGFβ). Patients with C2 and C3 subtypes were considered suitable for anti-TGFβ monoclonal antibody therapy. Patients with the C1 subtype had antitumor signatures, such as antitumor cytokines, effector cell traffic, M1 signature, MHCI, NK cells, and Th1 signature, resulting in a better prognosis after anti-PD-L1 mAb immunotherapy compared to patients with the C2 immune subtype. Branching trajectory analysis further identified the different suppressive phenotypes and the infiltration of immune cells in the tumor microenvironment by the Monocle package in R. The intragroup heterogeneity displayed no significant differences in overall survival, but there were significant differences in immune cell components and the tumor microenvironment signature in the four immune subtypes. Thus, the characterization of the immune landscape is critical for the prediction of the immune response to immunotherapy.

Previous studies have discovered that combinatorial biomarkers (ARID1A mutation plus CXCL13 expression) may improve prediction capability for urothelial carcinoma patients receiving immune checkpoint inhibitors (27), and IL6 may be the most promising predictive biomarker of peptide vaccines for colorectal cancer (28). In the present study, the WGCNA indicated that highly expressed genes in the black and pink modules were a responsive signature for immunotherapy, and three genes (FGFR1, ANXA6, and TGFB3) may be potential biomarkers for screening patients who are suitable for immunotherapy. Furthermore, a low expression level of TGFB3 was associated with complete or partial response to anti-PD-L1 monoclonal antibody treatment in urothelial carcinoma, resulting in a good prognosis. Inhibition of the TGF-β signaling pathway overcomes resistance to PD-1/PD-L1 blockade in cancer (29). The inhibition of TGFB3 in urothelial carcinoma may be a potential target to overcome immunotherapy resistance. In addition, although we characterized the immune landscape of the tumor microenvironment, more experiments and clinical exploration are still required for further validation.

## Conclusions

In summary, immunotherapy may be beneficial for patients with the C1 subtype. Hence, the present study provided a bioinformatics basis for understanding the immune landscape of the tumor microenvironment of urothelial carcinoma.

## Data availability statement

The original contributions presented in the study are included in the article/**Supplementary Material**. Further inquiries can be directed to the corresponding author.

## Ethics statement

Ethical review and approval was not required for the study on human participants in accordance with the local legislation and institutional requirements. Written informed consent for participation was not required for this study in accordance with the national legislation and the institutional requirements.

## Authors contributions

MP designed the study, performed bioinformatics analysis, prepared the figures, analyzed the data and interpretation, wrote and revised the manuscript.

## Funding

This study was supported by Natural Science Foundation of Hunan Provincial (2022JJ30831) and Natural Science Foundation of Changsha City (kq2202389).

## Acknowledgments

We thank TCGA (https://portal.gdc.cancer.gov/), ArrayExpress (https://www.ebi.ac.uk/arrayexpress/), European Genome-phenome archive (https://ega-archive.org/) and IMvigor210CoreBiologies (http://research-pub.gene.com/IMvigor210CoreBiologies/) for data collection for the provision of data processing. We also thank American Journal Experts (AJE) for English editing.

## Conflict of interest

The author declared that the research was conducted in the absence of any commercial or financial relationships that could be construed as a potential conflict of interest

## Publisher’s note

All claims expressed in this article are solely those of the authors and do not necessarily represent those of their affiliated organizations, or those of the publisher, the editors and the reviewers. Any product that may be evaluated in this article, or claim that may be made by its manufacturer, is not guaranteed or endorsed by the publisher.

## Supplementary material

Supplementary Figure 1 The different proportions of top10 high mutated genes in each immune subtypes were displayed.

Supplementary Figure 2 The immune cell infiltration in each immune subtypes using multiple algorithms.

Supplementary Figure 3 Validation of immune cell components and tumor microenvironment signatures in two other cohorts. **(A)** Immune cell components in the four distinct immune subtypes based on the E-MTAB-4321 cohort. **(B)** Immune cell components in the four distinct immune subtypes based on the IMVigor210 cohort. **(C)** Tumor microenvironment signature features in the four distinct immune subtypes based on the E-MTAB-4321 cohort. **(D)** Tumor microenvironment signature features in the four distinct immune subtypes based on the IMVigor210 cohort.

Supplementary Figure 4 Immune landscape of bladder urothelial carcinoma. **(A)** Tumor microenvironment signature in different subgroups of the C1 subtype. **(B)** Tumor microenvironment signature in different subgroups of the C2 subtype. **(C)** Tumor microenvironment signature in different subgroups of the C3 subtype. **(D)** Tumor microenvironment signature in different subgroups of the C4 subtype.

Supplementary Figure 5 GO and KEGG analyses in different modules. **(A)** Cellular component and molecular function in terms of the black module. **(B)** Cellular component and molecular function in terms of the pink module. **(C)** Cellular component and molecular function in terms of the red module. **(D)** KEGG signaling pathways in the black module. **(E)** KEGG signaling pathways in the pink module. **(F)** KEGG signaling pathways in the red module.

Supplementary Figure 6 The prognostic value and immunotherapy response of hub genes. **(A)** Kaplan–Meier overall survival curves for FGFR1. **(B)** Kaplan–Meier overall survival curves for ANXA6. **(C)** Kaplan–Meier overall survival curves for TGFB3. **(D)** The different expression of hub genes in different immune response groups.

## Abbreviations

TCGA, The Cancer Genome Atlas; BLCA, Bladder cancer; TME, tumor microenvironment; OS, Overall survival; WGCNA, Weighted Correlation Network Analysis; DDRTree, Discriminative dimensionality reduction with trees; TMB, Tumor mutational burden; TPM, transcript per million; ICPs, Immune checkpoints; ICD, Immunogenic cell death; MDSC, Myeloid-derived suppressor cells; DAMPs, Damage-associated molecular patterns; GO, Gene Ontology; KEGG, Kyoto Encyclopedia of Genes and Genomes.
