# Supplementary figures and images for: Immune landscape of distinct subtypes in urothelial carcinoma based on immune gene profile
Source: Front Immunol. 2022 Aug 8;13:970885. doi: 10.3389/fimmu.2022.970885 (PMC9394485; doi:10.3389/fimmu.2022.970885)

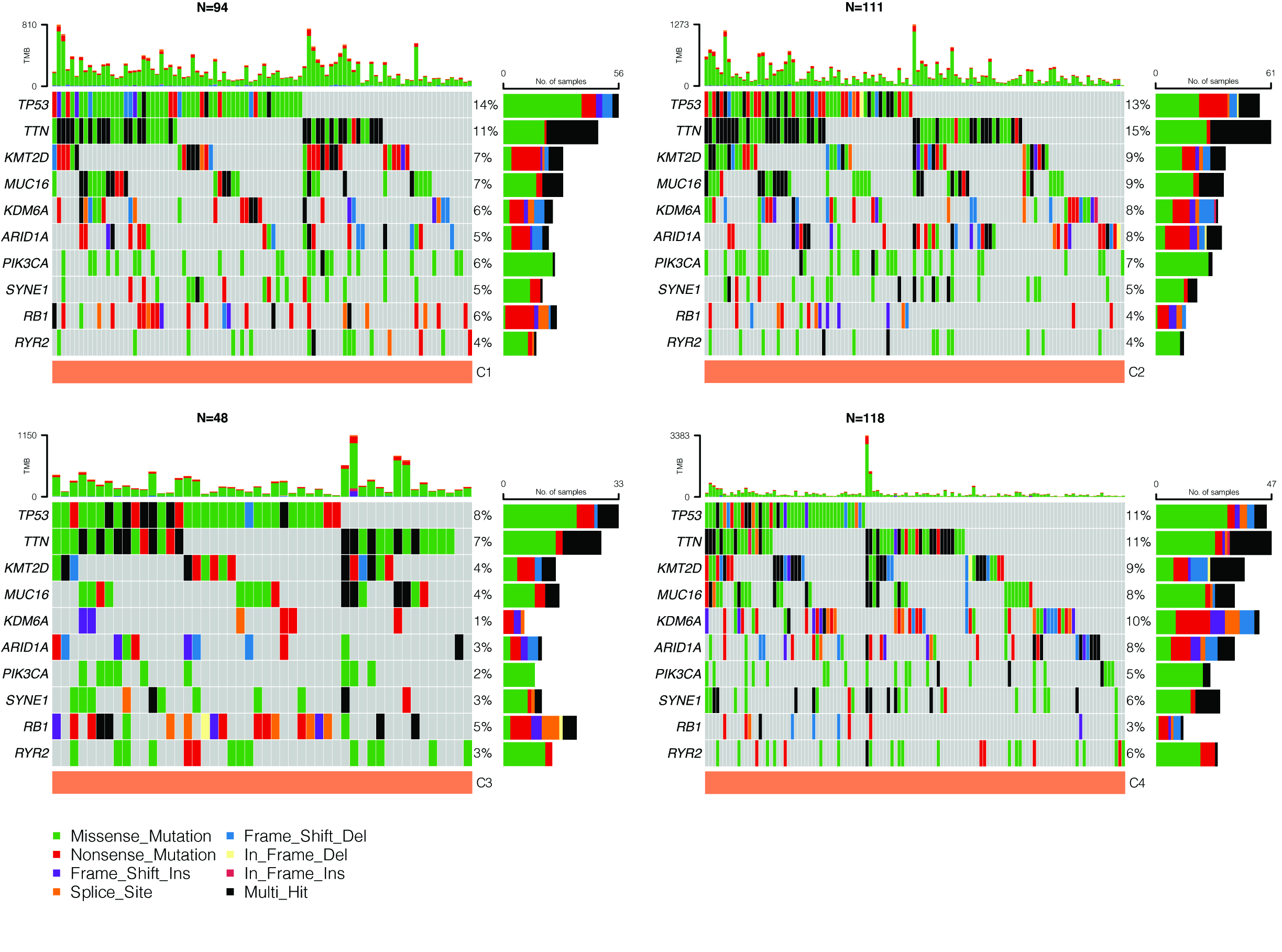

Supplement: Supplementary Figure 1 — The different proportions of top10 high mutated genes in each immune subtypes were displayed. [file Image_1.tif]

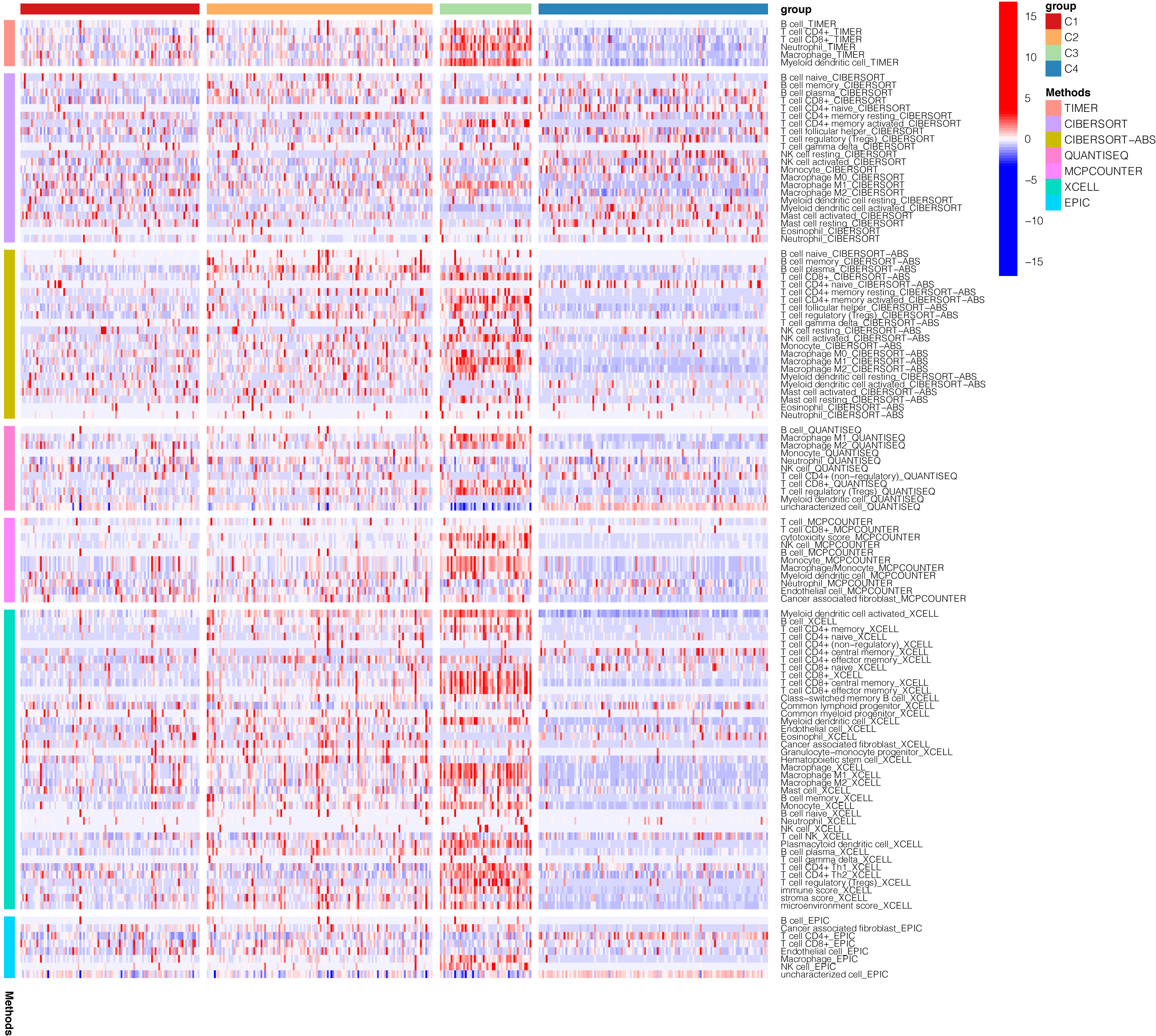

Supplement: Supplementary Figure 2 — The immune cell infiltration in each immune subtypes using multiple algorithms. [file Image_2.tif]

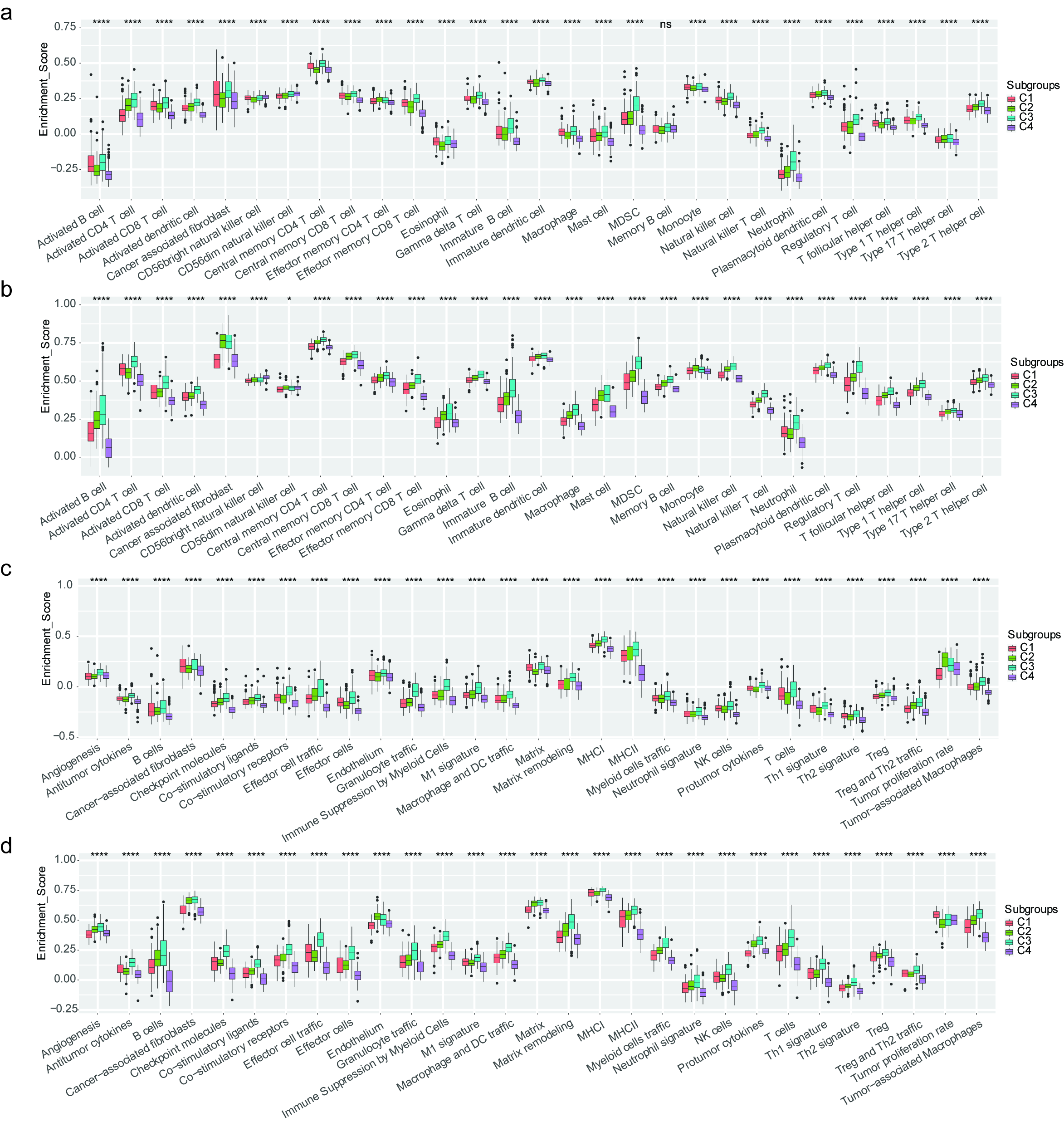

Supplement: Supplementary Figure 3 — Validation of immune cell components and tumor microenvironment signatures in two other cohorts. (A) Immune cell components in the four distinct immune subtypes based on the E-MTAB-4321 cohort. (B) Immune cell components in the four distinct immune subtypes based on the IMVigor210 cohort. (C) Tumor microenvironment signature features in the four distinct immune subtypes based on the E-MTAB-4321 cohort. (D) Tumor microenvironment signature features in the four distinct immune subtypes based on the IMVigor210 cohort. [file Image_3.tif]

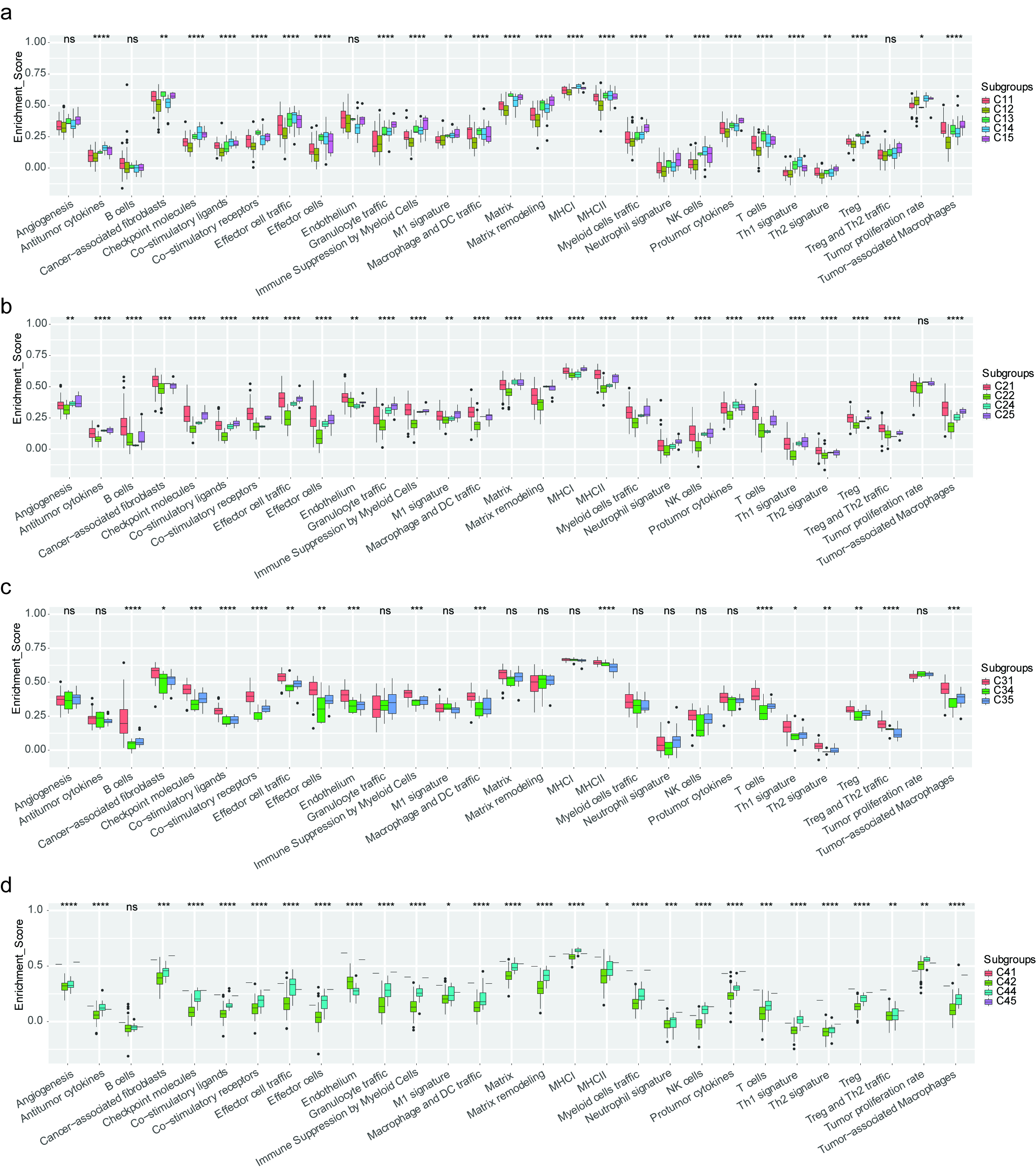

Supplement: Supplementary Figure 4 — Immune landscape of bladder urothelial carcinoma. (A) Tumor microenvironment signature in different subgroups of the C1 subtype. (B) Tumor microenvironment signature in different subgroups of the C2 subtype. (C) Tumor microenvironment signature in different subgroups of the C3 subtype. (D) Tumor microenvironment signature in different subgroups of the C4 subtype. [file Image_4.tif]

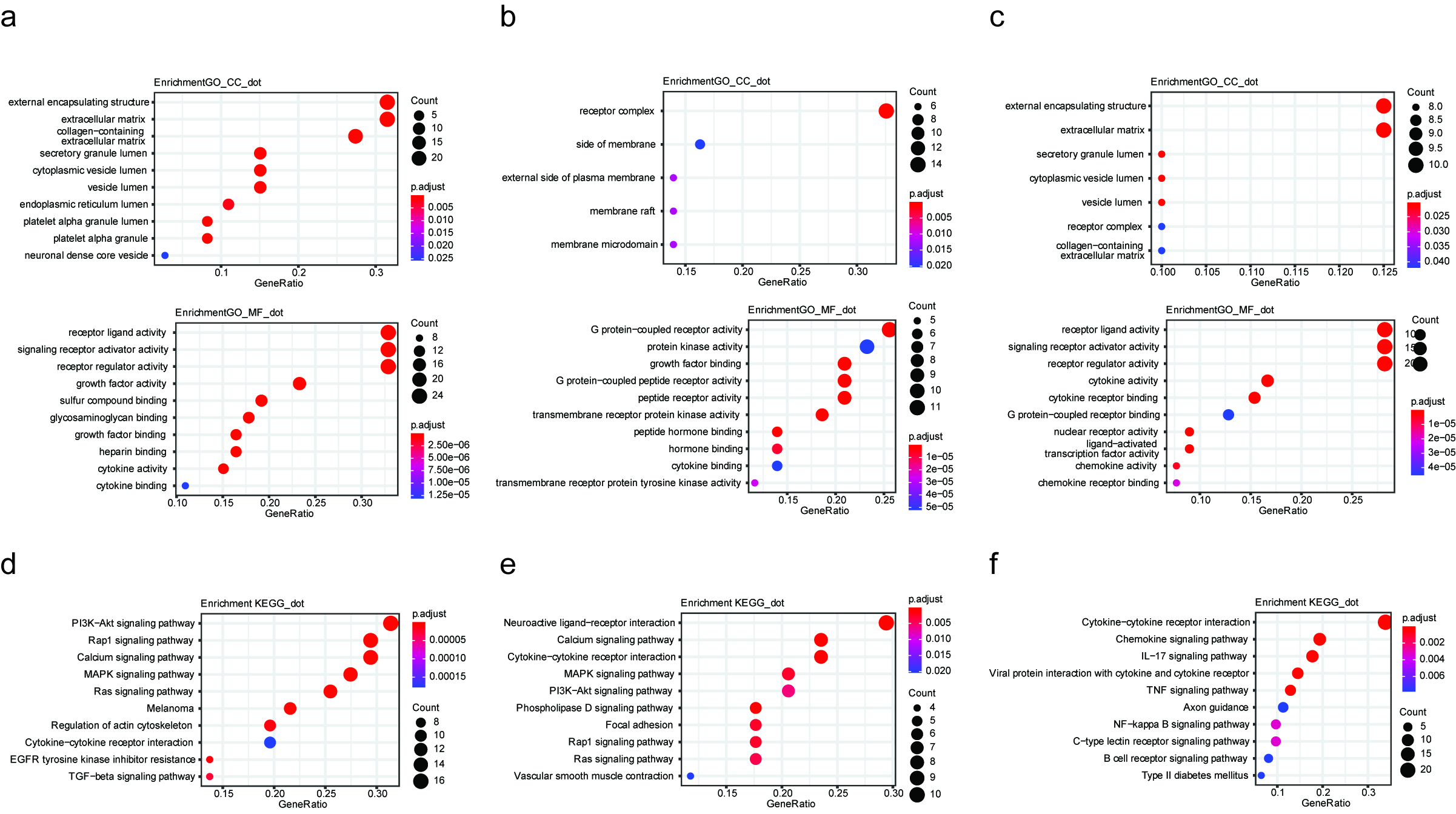

Supplement: Supplementary Figure 5 — GO and KEGG analyses in different modules. (A) Cellular component and molecular function in terms of the black module. (B) Cellular component and molecular function in terms of the pink module. (C) Cellular component and molecular function in terms of the red module. (D) KEGG signaling pathways in the black module. (E) KEGG signaling pathways in the pink module. (F) KEGG signaling pathways in the red module. [file Image_5.tif]

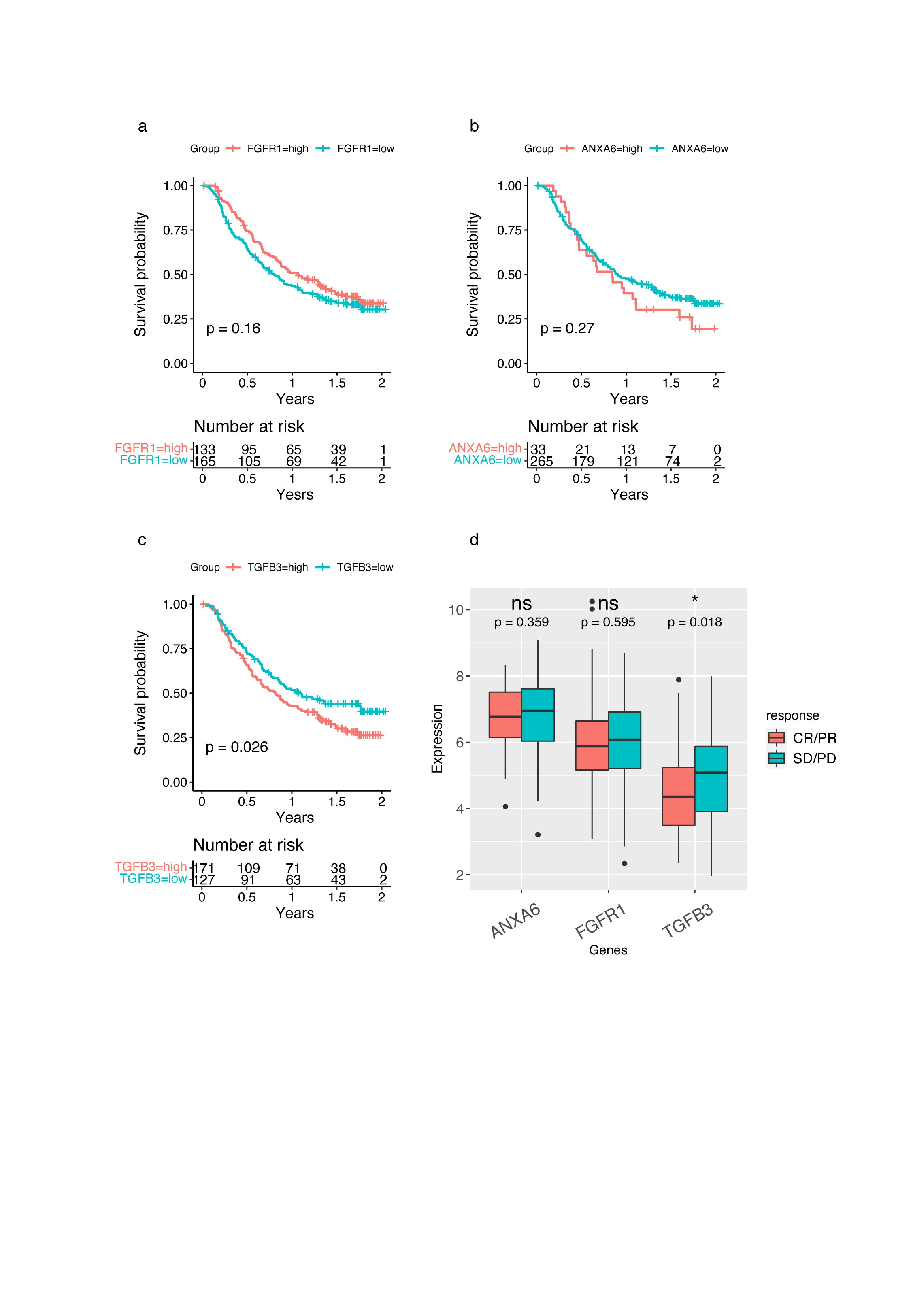

Supplement: Supplementary Figure 6 — The prognostic value and immunotherapy response of hub genes. (A) Kaplan–Meier overall survival curves for FGFR1. (B) Kaplan–Meier overall survival curves for ANXA6. (C) Kaplan–Meier overall survival curves for TGFB3. (D) The different expression of hub genes in different immune response groups. [file Image_6.tif]
